# Our Wished‐for Responses: Recommendations for Creating a Lived and Embodied Sense of Safety During Mental Health Crisis

**DOI:** 10.1111/hex.14122

**Published:** 2024-06-19

**Authors:** Helena Roennfeldt, Bridget Elizabeth Hamilton, Nicole Hill, Calista Castles, Helen Glover, Louise Byrne, Cath Roper

**Affiliations:** ^1^ Centre for Mental Health Nursing, Department of Nursing University of Melbourne Melbourne Australia; ^2^ Department of Social Work University of Melbourne Melbourne Australia; ^3^ Menzies Health Institute Queensland Griffith University Brisbane Australia; ^4^ Centre for Disability Research and Policy (CDRP) University of Sydney Melbourne Australia; ^5^ School of Management RMIT University Melbourne Victoria Australia; ^6^ Program for Recovery and Community Health, Department of Psychiatry Yale School of Medicine New Haven Connecticut USA

**Keywords:** crisis, crisis alternatives, crisis phone lines, crisis services, emergency department

## Abstract

**Background:**

Medical interventions have a place in crisis support; however, narrow biomedical and risk‐driven responses negatively impact people seeking crisis care. With increasing shifts towards involving people with lived experience (service users) in designing services, foregrounding people's desired responses is critical. Accordingly, the aim of the study was to explore the wished‐for crisis responses from the perspective of people who have experienced crisis and accessed crisis care.

**Method:**

Using a hermeneutical phenomenological approach, in‐depth interviews were conducted to determine the desired crisis responses of 31 people who self‐reported experiencing mental health crises and accessed crisis services at ED, phone lines and/or crisis alternatives.

**Results:**

The findings identified wished‐for responses that gave a felt and embodied sense of their own safety influenced by a human‐to‐human response, emotional holding, a place of safety and choice within holistic care. For such responses to be possible, participants identified organising principles, including recognising crisis as meaningful and part of our shared human experience, understanding risk as fluid and a whole‐of‐community responsibility for responding to crises.

**Conclusion:**

This paper proposes how insights from people who have experienced crises can be translated into more beneficial crisis care.

**Patient or Consumer Contribution:**

Most authors are in identified lived experience roles. The first author engaged with participants during the recruitment and interviews and was explicit regarding their lived experience. Service users were involved as advisors, providing input throughout the study.

## 1 Introduction

A mental health crisis has been described as an ‘acute disruption of psychological homoeostasis in which one's usual coping mechanisms fail, and there exists evidence of distress and functional impairment’ [1]. Throughout this paper, the term ‘crisis’ refers broadly to experiences of mental health crisis. Experiences of crisis are common and frequently require connection with others to resolve [2]. Increasingly, for those experiencing significant distress, the emergency department (ED) is the first or only point of contact for people voluntarily attending, referred to the ED or involuntarily transported to an ED for support [3, 4]. However, there is mounting evidence that the fast‐paced and highly biomedical environment within the ED is not appropriate or effective in responding to people in mental health crises [5].

People may also contact crisis phone lines (e.g., Lifeline), which constitute part of the broader public health strategy for responding to mental health crises and preventing suicide [6]. However, the evidence of the effectiveness of crisis phone lines is limited [7, 8]. In response to the mounting need for crisis care, there has also been interest in the development of community, peer‐based ED alternatives [9], such as the living room model [10] and crisis stabilisation centres [11]. The variety and number of ED alternatives have also increased in response to imperatives for greater involvement of people with lived experience in shaping more relevant service responses [12].

Understanding when someone is in crisis and what is helpful varies depending on the perspective of the individual experiencing the crisis, their family or the model of care used within crisis services [13]. From a clinician's point of view, an individual in crisis is assessed using standardised criteria and triage processes that prioritise responding to the risk of harm to self or others [14]. However, views from people accessing ED in a crisis frequently contradict those of clinicians on what help people require and when [5, 15]. Previous research asking service users what they wanted in crisis identified shared themes of accessibility, human connection and a holistic approach [16, 17].

The influence of lived experience perspectives has spawned reforms to maintain the intrinsic personhood of people seeking care [18]. Today, person‐centred and recovery‐oriented service delivery has been widely adopted within mental health policy and is central to mental health reform and better outcomes for people accessing services [19, 20]. Personal recovery recognises the critical role of people participating in decision‐making about their own lives [21] and their ability to discover personal meaning within their experiences [22]. For example, the Australian Safety and Quality Framework for Health Care sets out core principles for safety, including the need to be consumer‐centred and for safety to be achieved through shared decision‐making and learning from consumer experience [23].

Even so, translating the ideal of recovery‐oriented and person‐centred approaches to mental health practice has not sufficiently occurred [24]. This is evident in criticisms that have been raised regarding the use of physical and chemical restraint, involuntary treatment and involvement of emergency services (police and ambulance) within the ED environment, undermining a person‐centred approach [25]. Despite being widely accepted that the best approaches to mental health care are least restrictive practices and enhancing personal agency [26, 27], substantial changes are needed to translate these ideals into practice. Lived experience advocates have called for reforms to give greater autonomy and recovery‐oriented care that responds to the whole person and their social context, stating that crisis care ‘should not be traumatising, restrictive, disempowering, or burdensome’ [28].

Historically, within research, lived experience has been viewed as lacking validity and acceptability in the way evidence is gathered and constructed. Traditional evidence‐based measures separate ‘valid’ (scientific and rigorous) and ‘non‐valid’ (experiential, non‐rigorous) evidence [29]. However, over time, the Mental Health Consumer Movement, Mad Pride and Disability Rights have contributed to elevating the mental health consumer's voice and revalidating lived experience as a genuine form of knowing [29]. Nevertheless, the gap in what is considered valid forms of evidence remains. Lived experience accounts are integral to informing crisis service reform. However, there is limited research that provides expertise from lived experience guiding system transformation and translation to practice. Accordingly, the study aimed to explore the wished‐for crisis responses using a phenomenological study that privileged embodied and first‐person accounts of people who have experienced crisis and accessed crisis care.

## 2 Method

### 2.1 Study Design

A hermeneutical phenomenological approach [30] was used to deeply explore participants' subjective experience of crisis and distinguish wished‐for responses during a crisis experience. The study was approved by the University of Melbourne Human Research Ethics Committee. The potential for distress in discussing experiences of mental health crisis and crisis care was discussed with participants prior to the interview. Together, the researcher and participant discussed support needs and ensured that participants had access to a support person/service during and after the interview. A debriefing form was also provided to participants with contact details of online/phone services that participants can contact for additional support.

### 2.2 Participants

Recruitment was initiated via email and social media to Australian mental health Consumer and Peer networks. The inclusion criteria for participation were as follows: (a) self‐identified as having experienced a mental health crisis and (b) in response to this crisis, sought support at an ED, crisis phone line or alternative crisis service. Potential participants were contacted to confirm they met the inclusion criteria. Purposeful selection of study participants (over the age of 18) was undertaken to attempt to gain a representation of different crisis care experiences and recruitment continued until there was representation of people who had received crisis care across ED, crisis phone lines and crisis alternatives.

### 2.3 Procedure

In‐depth interviews focused on participants' embodied experiences of what was important within crisis responses to uncover personal and collective understandings of desired crisis responses. The first author conducted most interviews via the online Zoom video platform (*n* = 27), with two interviews by phone and two in person; the mean duration was 80 min (SD = 42). Interviews were audio‐recorded, de‐identified, and transcribed verbatim.

An initial broad question was asked to elicit a participant's narrative: What was your crisis experience that led to your encounter with crisis services? This initial question was followed by a question regarding the experience of crisis care: Can you describe the point where you received care services and what mattered most to you during this time? After that, participants were asked, ‘What would you have wanted in crisis?’ Responses such as being ‘listened to’ were explored in depth, appreciating that the experience of being listened to may mean different things to different people.

### 2.4 Analysis

The analysis was guided by Finlay's phases of Phenomenological Hermeneutic Interpretation [31]. These four phases consist of naïve understanding, structural analysis, comprehensive understanding and formulating the findings. During the analysis, the examination moved between data and researcher reflections, ensuring that the analysis stayed true to the words and meaning of the participants while being open to the researcher's reflections.

Analysis of the interviews was conducted by the first author, with discussion and review by the second and third authors. Interviews were analysed by following an in‐depth process of becoming familiar with the transcripts, writing a narrative summary of each interview, conducting an individual analysis and identification of themes of each interview, comparing themes across interview transcripts, clustering and refinement of themes, using ATLAS—ti Scientific Software Development GmbH (2022). In line with centring lived experience and a more in‐depth process of member checking [32], participants were provided with a copy of their narrative summary to confirm the accuracy and check quotes used in publications. Two participants provided edits to elements of their experience that could compromise their privacy.

The first author's lived experience of crisis gave an insider understanding, which was used transparently at the start of the interview and viewed as increasing empathy and trust [33]. Insider research, conducted by someone who shares a common experience, language and/or identity with research participants, can facilitate rapport building, enable more open engagement and produce more in‐depth data. Insider research can also be a vehicle for epistemic justice and emancipation, particularly in phenomenological research, because the voices of those with lived experience are prioritised over a priori theory or ontology [34].

## 3 Results

### 3.1 Participant Characteristics

The final sample was 31 out of 36 people who expressed an interest in participating; two people were ineligible, one decided not to participate and two did not respond when later contacted to arrange the interview. This sample size is consistent with recommendations for exploratory, qualitative interviews with a heterogeneous sample [35].

The participants identified as female (*n* = 20), male (*n* = 7), non‐binary (*n* = 2) and transgender (*n* = 2). The mean age was 42 (SD = 13). Most participants identified as having accessed ED (*n* = 30), and the number of presentations ranged from once to over 100 times; 25 participants used crisis phone lines, and 15 had accessed crisis alternatives and the period since participants had accessed crisis services ranged from within the last 6 months to over 5 years. Specifically for participants who accessed ED, seven participants accessed ED in the last 12 months, three in the past 2 years, 14 in the past 5 years and six longer than 5 years. The types of crisis alternatives participants had accessed covered formal ED alternatives such as Safe Spaces, which employed peer workers alongside clinical staff, peer‐operated services and a private hospital that could adopt more alternative and holistic approaches. For participants, the crisis experience was described as self‐injury, suicidality, psychosis and overwhelming emotions resulting from grief, past trauma or other life situations. Depending on their stated preference, the findings identify participants by pseudonym or their real name.

### 3.2 Wished‐for Responses

Participants included their desired responses within their accounts of crisis care. Overwhelmingly, these responses centred around establishing a felt sense of safety that emerged through a human‐to‐human response, choice within holistic care, a place of safety and emotional holding. Participants also identified organising principles that symbolised core beliefs underpinning wished‐for responses, including understanding crisis as part of the human condition; perceiving the crisis as meaningful; viewing risk as fluid; and acknowledging a whole community responsibility for the crisis. They suggested systemic changes were needed for crisis care to accord with these principles and their beliefs. The desired responses are discussed first, followed by the organising principles and an illustration is provided to bring these together.

### 3.3 Felt Sense of Safety

A felt sense of safety was the overarching requirement for people seeking crisis care and framed all other desired responses. Safety was embodied and emerged from a human‐to‐human response, emotional holding, choice within holistic care and a place of safety.

#### 3.3.1 Human‐to‐Human Response

A human‐to‐human response was essential in providing a sense of safety and was characterised as the quality of physical presence of others and not feeling alone in times of crisis.

Having more social support seems absolutely necessary because the current model of supporting people in crisis is to leave them to their own devices as if isolation is a good thing for people. Why would you think that isolation is appropriate? And I'm not talking about seclusion. I mean, like just being by themselves.

(Andy)

For participants, responding to someone in crisis involved offering care and safety through relationships by validating their emotional experience without an agenda of trying to ‘fix’ them.

The value of sitting with someone where they're at, yeah, is so much more weighty. Don't try to fix someone or to try and suggest this is how you should do life. When that respect is shown for who they are and where they're at, and sitting in the muck sometimes, but making sure they're not alone in that muck.

(Justin)

Peer workers were considered one way to address the need for a deeper connection that was not currently possible in ED.

I know they're going to be busy and under the pump in ED but what's stopping them from having a peer support worker in there? Let someone else go in that's not clinical because I think that's what made a big difference to me. If they weren't clinical, I didn't have a problem, and you tend to let them know what's going on and what you're feeling more, and you speak to them because they have this more calming demeanour about them.

(Sav)

Some participants described this quality of presence as leaving them feeling heard and seen.

In the midst of crisis, I think what I wish I could have sought was someone who could be present with me to listen but also just to be with… It has struck me that a lot of the times when I'm really struggling, what a difference it makes when someone is just present with you in the pain. And the pain is more bearable when you've got a witness, and you can start to share some things.

(Blair)

A human‐to‐human response was crucial for a sense of being connected with another during the crisis and for expressing validation. Validation recognised the seriousness of the situation and highlighted the importance of people who are comfortable being present with the experience of emotional pain.

#### 3.3.2 Emotional Holding

Emotional holding was described as the need for emotional pain to be held or contained in a relational space of safety. Essentially, participants wanted a response linked to the skills and qualities of ‘being with’ someone in crisis that was felt as being held.

This is life stuff that needs to be held and contained safely. To be able to have it and have it released. Where we can't contain it for ourselves, we need somewhere or someone to be that holding space.

(Emme)

Participants depicted emotional holding as sensing the emotional and physical closeness of others, ‘It's the physical presence with other people. I think that's a big thing, is not feeling like it like you're an imposition in any way', Morgan. A relational space where their emotions could be witnessed, ‘I think it's somewhere where I could be emotionally free for a bit where somebody says, ‘Are you okay?’ And then actually listens and where I cannot be okay’ (Rae).

Emotional holding resulted in a sense of personal safety that emerged within a relational and dialogical process.

I needed maybe six, eight, 12 hours, 24 hours even to just work through and talk. Just talk it out with someone and process. Kind of have the crisis be contained.

(Cathy)

Notably, emotional holding contrasted with physical containment and coercive approaches.

At its simplest, it's probably a human being present and knowing that they're not going to do things to you that you don't want. Yeah, I think there's something that could be powerful about that, just to be human with you and acknowledge that, you know, that they're human and that this is a human experience.

(Blair)

Emotional holding gave participants a feeling of release and being more comfortable in their skin. They felt emotionally contained in a way that allowed the crisis to be expressed within the relational space of a human‐to‐human connection. Emotional holding was valued as foundational to creating a sense of safety. Their desired crisis response required the experience of the crisis to be welcomed and acknowledged as a human experience. Importantly, a safe response was non‐coercive.

#### 3.3.3 Choice Within Holistic Care

Holistic care included the option of alternatives and the ability to access clinical care if needed or wanted.

Clinical care should play a part, I think, holistically speaking. I just feel like there's a lot of other roles that could be just as helpful. And just having a clinical focus by itself poses a problem, but I think that clinical definitely has a place.

(Mae)

Participants recognised that there was a need for medical intervention to be available at times of mental health crisis.

We need to have something in that overlap for people who are in mental health or suicidal crisis but also need access to physical health and clinical support.

(Helen)

However, the lack of choice caused participants to experience bodily tension as they were denied what they desired and thought to be healing.

Yeah, the clash was palpable, you know. I recognise now like the hospital is not there to heal but to medicate and stabilise. And, for me to want and ask for alternative therapies… So, um, the conflict was there.

(Darcy)

Holistic care involves choice in approaches and alternative therapies that respond to diverse worldviews and mental models of crisis. This meant that there would be more therapeutic options and broader consideration of other roles, such as psychologists, social workers and peer workers, in responding to emotional needs. Participants recognised the need for healing, not just stabilisation and the tension in the desire for alternative approaches, including acupuncture and body‐centred therapies, and being offered a narrow biomedical treatment.

#### 3.3.4 A Place of Safety

A place of safety was described as a physical place of welcome. For participants, this was somewhere to go that would not exclude them and where they could experience a sense of belonging.

Where you can sort of bring your whole self where you don't have the exclusion criteria everywhere.

(Helen)

A place of safety was seen to counter the perception that there is currently no place where people feel like they ‘fit’ and feel welcomed.

They've got no place for you. There's no place for you. So, it's just a place that I feel included and respected…where someone says, ‘This is a place for you’. It's not, you know, a square peg being fitted into a round hole.

(Justin)

A place of safety also included the desire for an alternate physical space that was homely and non‐clinical and reflected the wish for the crisis experience not to be viewed as a function of disease.

So, you feel like you're just in a place that's warm and welcoming, humane, and safe and not like a place that's busy and overly stimulating and also places where you have freedom and autonomy as well. And you feel like you have some control, and you feel like you're like you're not boxed in, and you have privacy.

(Cathy)

For people who had experienced forced treatment, having a place of safety within the service response that offered sanctuary was critical, ‘A sanctuary environment that is safe and free from traumatisation. You can't have a safe or healing service that does harm to people’ (Andy).

The characteristics of the physical environment were part of creating safety, but safety was also felt in knowing that there was somewhere to go and, in contrast to the ED, it was a place where they would feel welcomed and accepted. The need for privacy and a place of sanctuary was striking against the absence of safety and autonomy that was felt by people accessing standard crisis care. In short, the desire for crisis responses that gave a felt sense of safety was reflected in the wish for a human response, choice and a place that was healing and not harmful. Significantly, safety was experienced as embodied physical, emotional, relational and subjective that could not be ascribed externally.

### 3.4 Organising Principles Underpinning Wished‐for Responses

In addition to the wished‐for responses, participants identified organising principles that were necessary to enable the translation of desired crisis care into practice. These consisted of recognising crisis as part of the human condition, understanding crisis as meaningful, having a whole community responsibility for the crisis and recognising that risk is fluid. These core beliefs about the nature of the crisis were needed to ensure that desired responses were able to be translated into practice.

#### 3.4.1 Crisis as Part of the Human Condition

Behind the desire for alternatives and a human response to crisis was the need to view the crisis as part of the human condition and challenge the biomedical model's dominance.

Until crisis systems like de‐psychiatrise, it's going to be really hard for them to imagine anything else. We'll just see everything as a nail. I don't think psychiatry is a useful thing for people in crisis beyond the obvious fact that you can give people pills to make them calm down. Like that's a truth. But I don't think that's particularly useful. And that's not. I don't think that's a strong enough thesis for it to be the rationale for health services.

(Andy)

Changing the status quo also meant viewing experiences outside of psychiatry and changing how we name experiences.

But if they could stop saying anxiety and say someone's scared, clinicians actually do know what to do if someone feels scared. If they could stop seeing, you know, self‐injury and say, I'm simplifying here, but you know, say shame, and see someone who feels like they're not a worthy human being.

(Blair)

Crisis as a universal part of the human condition opened possibilities of responding to crisis outside of a biomedical approach, and based on shared human experience, ‘I would hope that people can drop the pretense of, like, the base state being okayness. Or, like, being without these feelings’ (Morgan).

This also included more open discussions about our common feelings to lessen peoples' experiences of difference and shame.

I see the only way that we can actually make a difference with all this is to address the stigma and the shame of feeling bad or feeling sad, feeling inadequate, feeling not good enough, feeling that the only way out is to kill ourselves. The shame and the stigma are so huge.

(Clare)

Moving to see crisis differently involved changing our language and being open to removing pathology regarding the crisis and reducing feelings of shame. Instead, it was proposed that what is needed is to recognise the experience of crisis as part of our human condition.

#### 3.4.2 Crisis as Meaningful

As part of our human condition, participants wanted the time of crisis to be a point where they could engage with others in a process of meaning‐making.

Time to come to my own understanding of what's happening. I didn't recognise as much as the people on the outside. I didn't recognise the DV because you get sucked into this vortex slowly, slowly, slowly. Yeah, I needed time and just being able to stop, think and let things evolve and see what I'm feeling.

(Di)

This reflected crisis as a meaningful experience rather than de‐contextualised as a form of pathology.

I think they see our distress as being a function of disease. And so, they don't see any reason, they don't see that it's meaningful. And while they don't see that it's meaningful, they don't see any reason to respond to it.

(Blair)

Generally, wished‐for crisis care was marked by responses that understood crisis as meaningful and favoured ways to facilitate meaning‐making.

#### 3.4.3 The Whole of Community Responsibility for Crisis

The need for a human response was related to the experience of crisis as part of the human condition and calls for societal change that accepts a whole of community accountability to respond to people in crisis.

I think we've all got a responsibility as human beings and should not be scared of people in crisis and push them off into a system that actually can do more harm than good. It seems to be, you know, in looking at the history that we used to feel a little bit more responsibility and, you know, some comfort and some confidence in being able to reach out and provide support to people in crisis, but gradually, it's just becoming more and more within the realm of mental health and outsourcing responsibility. Outsourcing to a big cold system.

(Blair)

As part of a whole‐community response, some participants were adamant that the government's role as a representative of the community was to provide support, and they wanted the government to step up and accept responsibility for supporting people.

If the government does not feel the responsibility to support people who are trauma survivors or support people who are mentally ill, then it is totally, like, a morally bankrupt government. Like the government is supposed to serve people and care for them, if it doesn't, the government almost has no right to exist. I do, I rely on the government to try and provide something for me. Because it's there for the people, and the government should try and provide services which actually meet the needs of people rather than just services existing based on precedent. So, it's not even a precedent. It hasn't even existed for that long. It's just how it ended up, which is unfortunate.

(Andy)

It was significant in relation to current dominant responses to the crisis in ED that participants recognised a foundational need for social change for alternative crisis responses to be established and supported within the community. There was an identified whole of community and government responsibility to care for people in crisis.

#### 3.4.4 Risk Is Fluid

Risk was perceived as fluid and influenced by relationships and context. For participants, safety was seen as accepting risk and that it is not possible or helpful to remove all risk.

A safe space accepts that requirement of risk… risk was not accepted by the hospital. The risk was all removed. Yeah, it was sterilised. So, it actually increases some risk in other areas. But, you know, we're gonna make sure he can't kill himself because we're gonna put him in a room that he can't kill himself in, and then we're gonna put him under medication, and then we're gonna put him in a mental health unit, and we're gonna wash our hands of the problem.

(Gregory)

Understanding risk validates the seriousness of the crisis experience and the potential for suicide.

Those courageous conversations about, yes, I understand from the medical‐legal perspective that you want to put me in the hospital right now. But this is actually going to cause more damage, and I would rather be dead than be in hospital.

(Addison)

**Figure 1:**
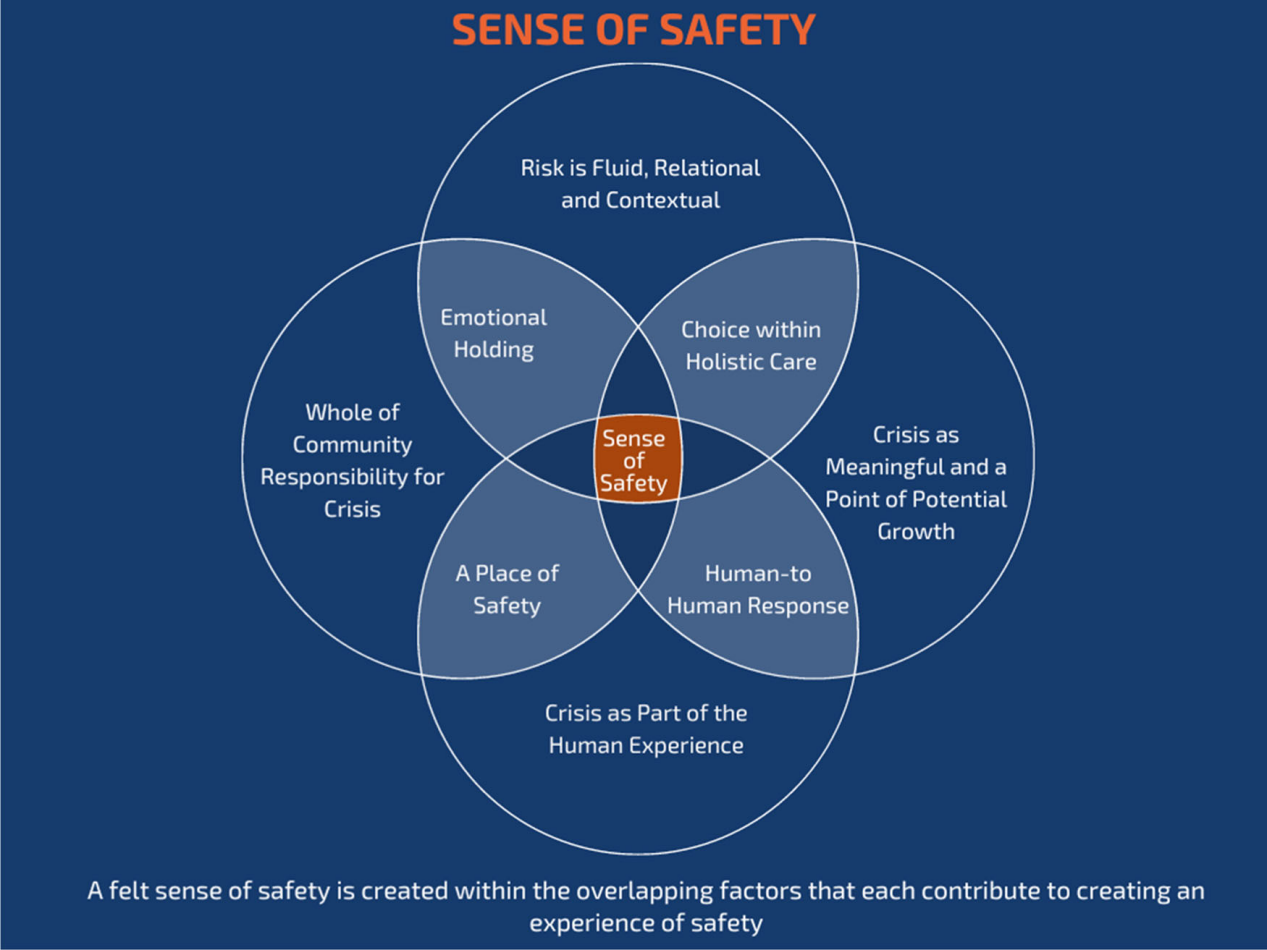
Conditions for creating a felt sense of safety: an interplay of desired responses and organising factors.

Accepting risk involved choice and control for people in crisis and finding ways to create safety that were non‐coercive and free from traumatisation. Participants outlined organising principles necessary to drive the shift towards creating the conditions needed to create an embodied sense of safety. Together, the desired responses and organising principles are presented graphically here to show the complex interplay between these elements to create a sense of safety, as shown below in Figure 1.

Ultimately, the organising principles represented a paradigm shift away from pathologising crisis and towards understanding it as part of the human condition, regardless of whether it was named a mental health, suicidal or situational crisis.

## 4 Discussion

This study explored the wished‐for responses of people who had received crisis care in ED, crisis phone lines and crisis alternatives. Overall, a felt sense of safety was the thread that tied together participants who desired crisis care. A felt sense of safety was achieved through a human‐to‐human response, emotional holding, holistic care and a welcoming place of safety. In previous research, a sense of safety is conceived within a person's context and relationships as a shift towards a more whole‐person approach to health, healing and growth [36]. Similarly, participants identified safety as subjective and variable depending on internal appraisal and external environment. This means that external opinions of safety and risk assessments do not necessarily translate to the person feeling safe. There are ways of working with overwhelming emotional experiences; for example, Peter Levine has popularised the idea of felt sense in understanding the impact of trauma [37]. Equally, participants' lived experience forms the basis for knowing and understanding what is needed to create an embodied experience of safety.

The desire for a human response was based on the universality of crisis as part of the human experience, delineated from biomedical care based on pathology. Relatedly, wanting a human response overturns the socially constructed taboo and deviance attached to the experience of suicide [38]. Findings correspond with theories of suicide as an understandable way to escape pain [39]. Additionally, a human‐to‐human response speaks to the need to counter experiences of ‘othering’ that continue to emerge in research regarding mental illness and reflect the manifest pathology ascribed to a mental health crisis [40]. Findings support previous research and recommendations from lived experience advocates that identified mental health crises as part of our human experience and an understandable response to social factors [41, 42, 43]. Globally, guidance from the World Health Organisation (WHO) advocates for mental health policy and laws moving away from the exclusivity of narrow biomedical frames and coercive practices [44]. However, most countries have fallen short of challenging the dominance of the biomedical paradigm and fail to embrace a greater focus on a rights‐based approach within mental health services as recommended by the World Health Organisation: ‘Legislation on mental health must therefore take a new direction away from the narrow traditional “biomedical paradigm” that has contributed to coercive and confined environments in mental health services’ [44].

For participants, emotional holding was present in a connection where overwhelming emotions could be expressed and held. This reflected a need for a relational response built on empathy and trust to remove the need for more physically restrictive ‘measures of control’. The understanding of emotional containment demonstrated that being held within a relational response to crisis results in a felt sense of safety, and there is evidence that therapeutic relationships can provide emotional holding and safe relational space [45].

The desire to choose treatment options and non‐coercive care was fundamental to achieving a sense of safety. Participants wanted support to be readily accessible but not forced. Involuntary care is based on a clinical assessment of the risk of suicide or harm to others that is known to carry risks of traumatisation [46]. This was supported in the findings, and instead, participants wanted to engage in a shared understanding of risk where their experience was heard and respected. There was a wish for the emotional pain to be witnessed and for the seriousness of the risk these experiences posed for people's lives to be acknowledged, but not in a way that stripped people of self‐determination and personal agency.

A place of safety included an experience that countered feeling excluded and a desire for a homely physical space. This finding mirrors the evidence of the negative experiences of feeling like experiences of emotional distress and suicidality were delegitimised and dismissed when presenting to ED [5, 47]. Similarly, research has highlighted the desire to feel welcomed in crisis [48]. This finding also supports the well‐established need for a sense of community in supporting wellbeing [49]. Previous research has also shown that peer roles in mental health are effective in creating inclusion and reducing experiences of stigma [50, 51].

The findings also uncover organising principles that frame crisis as part of the human experience and having meaning, requiring a whole community response and a shared understanding of risk. Findings also align with what we know is needed in providing recovery‐oriented care [27] and care that supports safety [23]. Experiences of mental health crisis and suicidality were seen as deeply rooted within human existence and in the essential structure of being human. The crisis was viewed as a painful but meaningful experience and directly affected by our socio‐political drivers of distress. Participants identified thwarted potential for meaning‐making, highlighting known limitations of a narrow biomedical understanding of distress [52]. Likewise, commentators have previously warned against reductionist notions of ‘illness’ and crisis as something to be treated and eliminated rather than explored [53].

This research has asserted the consumer's voice and used lived experience perspectives to develop wished‐for crisis care. Lived experience is, by necessity, a valid form of embodied truth and collective accounts are used to identify consensus in desired responses. Desired crisis care involves implicit and embodied knowledge about what is needed during an experience of crisis that is not easy to grasp and describe. Crisis responses are also understood within a specific relational and physical context, and an understanding of wished‐for responses may be limited because people cannot always know what they have not experienced. The disclosure of the interviewer's lived experience may also have influenced what was shared in the interview. However, the validity of the research is strengthened by the additional member checking and confirmation from participants. Phenomenology and elevation of the consumer's voice in rigorous research processes are recommended as part of the canon of evidence used in developing crisis care.

### 4.1 Summary of Recommendations for Crisis Care

Understanding the wished‐for responses through the words of people with lived experience has much to offer crisis care reforms. Participants desired responses and organising principles suggest that the following recommendations may be valuable to the care of those experiencing a crisis. These include:

- Making peer support workers available in ED. Access to peer workers was thought to offer a more human approach that countered the dominance of a narrow biomedical perspective. Peer workers are increasingly known for providing effective recovery‐oriented and person‐centred care that recognises the transformational potential for crisis [54] and can help facilitate different framings and meaning‐making of experiences [43].

- Encouraging the telling of personal narratives as a growing search for meaning during crisis. Viewing crisis as part of the human condition is a way of non‐pathologising and understanding that crisis can hold meaning within a person's life. Existential approaches likewise recognise the ubiquity of crisis as part of the human condition and a meaningful, potentially transformative experience [44].
- Encouraging relational responses centred on connection, deep listening and being present with someone in crisis. These relational responses offer emotional holding and foster non‐coercive care represented by choice, autonomy and control. The concept of containing is based on Jung's alchemical container in which overwhelming thoughts and feelings are held in the process of *being with* [55].
- A place of safety and accessible support both before the point of crisis and when someone is in crisis recognising that there were points in the crisis timeline where people could intervene earlier before a point of absolute crisis. The principle of whole community responsibility for crisis recognises community and holistic care that includes alternatives to ED and allied health, such as psychologists and social workers within ED services. Equipping members of the community to respond to crises indicates that it is feasible to decentralise crisis away from primarily clinical service responses [56].
- Encourage a shared dialogue and understanding of risk, including potential iatrogenic harm in receiving crisis care. Shared decision‐making and a joint understanding of risk emphasise self‐determination and dignity of risk instead of relying on standardised service responses [57, 58]. There is also a focus on personal and collective responsibility [43]. Open Dialogue is one approach that holds promise as a democratic way for the person in crisis and those offering support to feel listened to and for differences in meaning to be respected through the principle of ‘tolerating uncertainty’ [59].
- Community response to crisis considers human crisis beyond mental health systems that can counter psycho‐centrism [43, 60]. The multilayered and collective nature of crises offers a political reconceptualisation of crisis, requiring a deliberate and paradigmatic definition of crisis that allows for individual interpretation of a crisis and when help is needed. Holistic and sociocentric models demonstrate the feasibility of working outside a medical model, including the Soteria paradigm [60], which features communal, social approaches to care.

Recommendations combine the desired responses with potential tangible changes needed for policy and practice. Essentially, they highlight the need for personalised support based on deep listening and holding space for someone in crisis. We need responses to crises that acknowledge the complex interplay of psychosocial, political and relational influences on human distress and recognise crisis as part of our human condition.

## 5 Conclusion

The effectiveness of crisis responses depends on whose voice is included in decision‐making. The subjective experience of people who have experienced crisis and their articulation of desired responses have largely been excluded. Knowledge from lived experience is vital in a context where what is valued is usually knowledge based on clinical outcome measures and external risk assessments. Lived experience knowledge becomes a counter‐discourse for clinical evidence‐based outcomes and challenges how crisis care is conceptualised. Findings and recommendations provide valuable insights for providers, policymakers and funders in providing meaningful responses to crises.

## Author Contributions

**Helena Roennfeldt:** conceptualisation, methodology, writing–review and editing, writing–original draft, formal analysis. **Bridget Elizabeth Hamilton:** supervision, writing–review and editing, formal analysis. **Nicole Hill:** supervision, writing–review and editing, formal analysis. **Calista Castles:** writing–review and editing. **Helen Glover:** writing–review and editing. **Louise Byrne:** supervision, writing–review and editing. **Cath Roper:** writing–review and editing.

## Conflicts of Interest

The authors declare no conflicts of interest.

## Data Availability

The data that support the findings of this study are available on request from the corresponding author. The data are not publicly available due to privacy or ethical restrictions.
